# Acute and Cumulative Effects of Repeated Exposure to Chlorpyrifos on the Liver and Kidney Function among Egyptian Adolescents

**DOI:** 10.3390/toxics9060137

**Published:** 2021-06-10

**Authors:** Ahmed A. Ismail, Olfat Hendy, Gaafar Abdel Rasoul, James R. Olson, Matthew R. Bonner, Diane S. Rohlman

**Affiliations:** 1 Department of Occupational and Environmental Health, College of Public Health, University of Iowa, Iowa City, IA 52242, USA; diane-rohlman@uiowa.edu; 2 Community Medicine and Public Health Department, Faculty of Medicine, Menoufia University, Shebin Elkom 32511, Egypt; gaafar237@yahoo.com; 3 Kansas Department of Health and Environment, State of Kansas, Topeka, KS 66612, USA; 4 Department of Clinical Pathology, National Liver Institute, Menoufia University, Shebin Elkom 32511, Egypt; olfat_hendy@hotmail.com; 5 Department of Epidemiology and Environmental Health, State University of New York, Buffalo, NY 14260, USA; jolson@buffalo.edu (J.R.O.); mrbonner@buffalo.edu (M.R.B.); 6 Department of Pharmacology and Toxicology, State University of New York, Buffalo, NY 14260, USA

**Keywords:** adolescents, liver function, kidney function, organophosphorus, chlorpyrifos, cholinesterase activity

## Abstract

Background: There is a paucity of research that tracks changes in liver and kidney function among pesticide applicators. The aim of the current study was to investigate the role of repeated seasonal exposure to the organophosphorus pesticide, chlorpyrifos, on serum measures of liver and kidney function. Methods: Pesticide exposure was assessed by measuring the urinary concentrations of 3,5,6-trichloro-2 pyridinol (TCPy), a specific biomarker for chlorpyrifos. Chlorpyrifos exposure and 8 serum markers of liver and kidney function were measured at 15 timepoints over 3 years prior to, during, and following the end of seasonal pesticide application among adolescent applicators and non-applicators from 4 field stations in Menoufia, Egypt. Results: Urinary TCPy levels showed increases during the application cycles and recovery at the end of each application season. Altered serum markers of liver and kidney function were associated with chlorpyrifos exposure, with some markers recovering 3 months after the end of exposure each year, while other measures demonstrated progressive increase up to 300% the baseline levels at the end of 3 years. Conclusion: The study demonstrated that frequent assessment of liver and kidney function is a sound practice to evaluate cellular injury following chronic repeated occupational and environmental exposure to chlorpyrifos.

## 1. Introduction

The liver is the organ where activation and detoxification of organophosphorus (OP) compounds take place, with the metabolites being eliminated primarily through the kidneys [1]. The kidneys receive about 20–25% of the resting cardiac output in the body. Thus, any drug or chemical in the systemic circulation will be delivered to the kidneys in relatively high amounts. The process involved in forming concentrated urine also serves to concentrate potential toxicants into tubular cells. Therefore, a nontoxic concentration of a chemical in the plasma may reach toxic concentrations in the kidney. The subsequent renal transport, accumulation, and metabolism of xenobiotics contributes significantly to the susceptibility of the kidney to toxic injury [2].

Chlorpyrifos (O, O-diethyl O-3,5,6-trichloro-2-pyridyl phosphorothioate (CPF)) is the main OP pesticide applied in Egypt to control cotton insects [3]. In humans, cytochrome P450 enzymes (CYPs) detoxify CPF to 3,5,6-trichloro-2-pyridinol (TCPy, urinary biomarker of exposure) and bioactivate CPF to chlorpyrifos-oxon (CPF-O), which is the metabolite responsible for the inhibition of β-esterases, including acetylcholinesterase (AChE), butyrylcholinesterase (BChE), and carboxylesterase. However, long-term exposure to CPF has been associated with a number of other adverse health effects through cholinergic and noncholinergic mechanisms [4]. Interestingly, Foxenberg et al. [5] found that human cytochrome P-450 2B6 (CYP2B6) is the enzyme primarily responsible for bioactivation of CPF to the active oxon metabolite, and Knights et al. [6] reported that CYP2B6 is on a few cytochrome-P450s expressed in the human kidney. Therefore, the liver and kidney have been investigated as potential target organs for the acute, sub-chronic, and chronic exposure of experimental animals to OP pesticides [7,8].

Repeated exposure to CPF may lead to the accumulation of toxins in the liver, which may result in pathological alterations in the liver cells [9]. These pathological alterations may present with increased permeability of plasma membranes or cellular necrosis, resulting in increased release of tissue specific enzymes into the blood [10]. Several studies have investigated changes in liver enzymes among workers exposed to pesticides, with the majority of these studies reporting elevated serum levels of Alanine Aminotransferase (ALT), Aspartate Aminotransferase (AST), and Alkaline Phosphatase (ALP) [11,12,13,14]. Conversely, other studies have found decreased liver enzymes [15,16]. Although Hu and colleagues reported increased levels of liver enzymes 3 days post-exposure, these changes were not observable at 4–10 days post-exposure [17], indicating recovery. Following a cohort of pesticide applicators who are seasonally exposed to pesticides for several years would assist in characterizing perturbations in liver enzymes over time.

Chronic kidney disease (CKD) of early onset among young men in agricultural communities is a growing public health concern. The disease is characterized by tubulointerstitial pathology without obvious risk factors, e.g., hypertension, diabetes, and aging [18]. CKD was first reported among workers in sugar cane plantations in Central America in the early 2000s [19,20] and was then described in other occupations, including cotton and corn workers [21], shrimp farm workers [22], construction workers [23], and mine workers [24]. Peraza and colleagues reported that cotton workers had a higher prevalence of elevated serum creatinine and decreased estimated glomerular filtration rate compared with residents of an urban community. The prevalence of elevated levels of serum creatinine was also higher among older compared to younger former cotton workers [21].

Few studies have examined repeated exposure to pesticides and renal function parameters among farmworkers [20,25,26]. Progressive renal dysfunction was observed in farmworkers repeatedly exposed to pesticides during crop planting seasons [20,25], as well as toward the end of the harvest season when they frequently presented with episodes of acute abnormal kidney function during the work shift [25]. Kupferman and colleagues also reported that workers with an increase in serum creatinine levels at the end of the harvest season did not fully recover during the following year, which led to CKD in one-third of the workers [20]. To explore the risk factors of CKD among farmworkers, wells in areas of Sri Lanka with a higher prevalence of CKD were examined for pesticides. It was found that CKD was common among individuals with a history of drinking from wells contaminated with pesticides, which might explain the presence of CKD even among non-working individuals [26].

The previously mentioned studies indicate the importance of employing sensitive liver and kidney biomarkers for early detection of liver and renal toxicity before the appearance of adverse clinical health effects as a result of exposure to OP pesticides [27]. However, none of the previous studies monitored changes in these function parameters across several pesticide application seasons to study the relationship with repeated exposure and whether these changes deteriorate or recover over time. The study research team utilized an established cohort of adolescent pesticide applicators and non-applicators to assess the changes in liver and kidney function during, immediately following, and 3 months after the end of the CPF application cycle over a 3-year period. CPF is the major pesticide applied to the cotton crop in this agricultural region of Egypt. The longitudinal study design provided a robust approach to assess alterations in serum makers of liver and kidney function associated with exposure to CPF.

## 2. Materials and Methods

### 2.1. Study Population and Setting

The study was carried out in Menoufia Governorate (The Delta of the Nile, Lower Egypt), where cotton is a strategic crop for the whole country. Adolescents are hired seasonally as applicators by the Ministry of Agriculture (MOA) to spray cotton fields with pesticides over a period extending from early June to late August. Applicators work in teams of 3–4 individuals with adult supervisors. Applicators assist with loading equipment and supplies and mixing and filling of backpack sprayers with pesticides. Then, they carry the backpack sprayers to apply the pesticides to the cotton fields. Although the MOA regulates the application process centrally all over the country, the type and duration of the applied pesticide depend on the degree of insect infestation in each cotton field. Generally, the pesticide application process occurs in 4 cycles starting with a biological growth stimulator, followed by an OP compound, primarily CPF, then a cycle of pyrethroid pesticides (e.g., α-cypermethrin or γ-cyhalothrin), and finally another cycle of CPF application. Application occurs daily in the afternoon for about 5 hours. In our experience with this applicator population, personal protective equipment (PPE) use is sporadic and inconsistent [28,29]. Nonetheless, we used urinary TCPy that estimates internal uptake of CPF and accounts for any PPE or other external factors that could influence internal dose.

**Figure 1:**
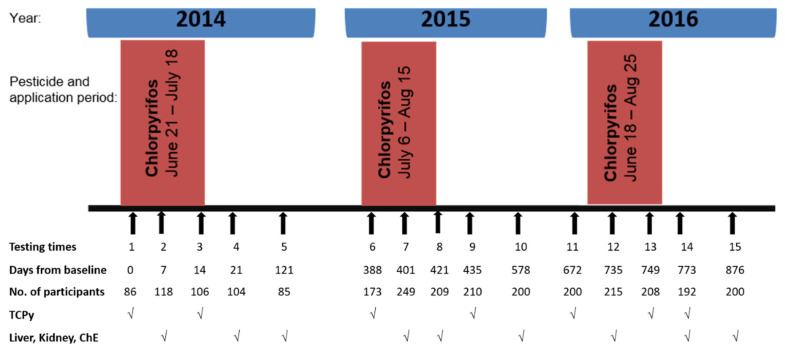
Chlorpyrifos application, days from baseline of the testing timepoints, number of participants, and availability of liver and kidney function measures and urinary TCPy measurements across the 3 years of study.

A longitudinal prospective study was conducted in 4 field stations (Quesna, Shohada, Tala, and Berket El-Sabe’) randomly selected from the 9 field stations in Menoufia Governorate (Egypt) to study various types of health effects of exposure to pesticides. All applicators in the 4 field stations were invited to participate in the study, and the response rate was 100%. For the current study, 12–21-year-old adolescent male applicators were recruited from the 4 field stations during 2014–2016, with 15 testing timepoints available for analysis, 5 times a year (Figure 1). In 2014, there were only participants from 3 stations (Quesna, Shohada, Tala). Starting in 2015, adolescents who never worked in pesticide application with the Ministry of Agriculture were also invited to participate. The non-applicators were relatives and friends of the applicators living in the same communities. Females do not apply pesticides for the Ministry of Agricultural (MOA) and were not included in the study. Although non-applicators did not work with MOA, they may have been exposed to pesticides during the application of pesticides at homes. Exclusion criteria included diagnosis of liver or kidney disease. However, none of the participants were found to have any of these diseases. The study was conducted according to the guidelines of the Declaration of Helsinki, and approved by the Institutional Review Boards of the University of Iowa (protocol code 201301760 on June 20, 2014) and Menoufia University (protocol code 50379 on June 22, 2014). Participants and their legal guardians gave written informed consent prior to enrollment. 

### 2.2. Procedures

As shown in Figure 1, study participants were examined 5 times each year, prior to, during, and after the application of CPF, the major pesticide applied to the cotton crop in this agricultural region of Egypt. Figure 1 also shows the CPF application period each year, days from baseline for each testing time (baseline was in July 2014), and the number of participants, as well as the available data at each of these timepoints. In 2014, a total of 119 applicators were recruited. In 2015, 102 (86.0%) of these applicators continued to participate and an additional 101 applicators and 76 non-applicators were enrolled. In 2016, 182 (90%) of applicators and 67 (88.0%) of non-applicators continued to participate in the study. 

The assessment of liver and kidney function occurred 3 times each year: Once during the CPF application (timepoints 2, 7, 12), once immediately following the CPF application (times 4, 8, 14), and once about 3 months after the end of the CPF application (times 5, 10, 15). Urinary levels of 3,5,6-trichloro-2 pyridinol (TCPy), a specific metabolite of CPF and sensitive biomarker of CPF exposure, were measured 7 times (1, 3, 6, 9, 11, 13, 14) across the 3 years of study.

### 2.3. Questionnaires

Both cohorts (applicators and non-applicators) of the study completed a baseline questionnaire that queried medical and occupational history, including detailed questions about exposure to pesticides. These questions collected information about residential pesticide application among both cohorts and exposure while working for the MOA, working as private pesticide applicators, and applying pesticides in family fields among applicators. In addition to the baseline questionnaire that was completed annually before the beginning of the application season, a short questionnaire was completed at each timepoint that asked about participants’ exposure that day, as well as their exposure during the previous week. The short questionnaire was completed by all participants. 

### 2.4. Blood Sampling

Blood samples from both study cohorts were collected at the field stations 3 times each year to examine liver and kidney function and cholinesterase activity during, immediately following, and about 3 months after the CPF application cycle (Figure 1). Venous blood samples were drawn from participants under complete aseptic procedures and divided into 2 parts: 1 part was put into an EDTA-coated vacutainer tube, and the remaining part was put into a vacutainer plain tube. The EDTA blood samples were immediately placed on wet ice and transported to Menoufia University where they were analyzed in duplicate for liver and kidney function, as well as for assessment of cholinesterase activity, acetyl cholinesterase (AChE), and butyryl cholinesterase (BChE). Serum samples were separated by centrifugation of the blood samples from plain tubes at 3600 rpm for 15 min in a refrigerated centrifuge at 4 °C. Serum samples were collected in 2 aliquots and kept at −20 °C until analysis for liver and kidney function. 

### 2.5. Cholinesterase (ChE) Analysis

The EDTA blood samples were analyzed in duplicate for AChE and BChE activity using an EQM Test-Mate kit (EQM Research Inc., Cincinnati, OH, USA) as described previously [30]. The method depends on the Ellman method [31] to measure cholinesterase activity among pesticide applicators and non-applicators employing a portable analyzer [32].

### 2.6. Analysis of Liver and Kidney Function

Liver and kidney function, including serum Alanine Aminotransferase (ALT), Aspartate Aminotransferase (AST), Alkaline Phosphatase (ALP), Gamma Glutamyl Transpeptidase (GGT), total bilirubin (TB), direct bilirubin (DB), blood urea, and creatinine were analyzed at the laboratories of the National Liver Institute, Menoufia University. Activities of ALP, AST, ALT, and GGT were determined using an auto analyzer (COBAS 601e-Roche Diagnostics). Other measures were estimated as follows: Serum bilirubin by photometric method based on the diazo reaction [33], urea by Diacetyl monoxime method [34], and creatinine by Jaffe’s alkaline picrate method [35]. All the liver function measures were conducted in whole blood, while creatinine and urea were assessed in serum.

### 2.7. Urine Collection and Analysis for 3,5,6-Trichloro-2 Pyridinol (TCPy)

Spot urine samples were collected from the study participants at each timepoint before starting their work shift. The collected samples were transferred on wet ice to the Menoufia University laboratories in Shebin El-Kom, Egypt. The samples were then stored at −20 °C until they were shipped on dry ice for analysis at the University at Buffalo (Buffalo, NY, USA). The urine samples were analyzed for the specific metabolite of CPF, 3,5,6-trichloro-2 pyridinol (TCPy), as explained in detail previously [30]. The analysis method included hydrolysis, extraction, derivatization, and analysis by negative-ion chemical ionization gas chromatography-mass spectrometry (GC/MS) utilizing ^13^C-^15^N-3,5,6-TCPy as an internal standard. Creatinine was colorimetrically analyzed using the Jaffe reaction [36], and urine TCPy concentrations are expressed as micrograms TCPy per gram creatinine. The precision of the analysis method was high, with a 0.997 intraclass correlation coefficient and 0.5 ng/mL detection level. TCPy levels from 7 timepoints across the 3 years of study are available for the purpose of this work (times 1, 3, 6, 9, 11, 13, 14).

### 2.8. Statistical Analysis

Statistical analysis was performed using SAS (version 9.3, SAS Institute, Cary, NC, USA). Bivariate analysis was conducted to assess the relationship between the different exposure characteristics and the liver and kidney functions. The main exposure metric was urinary TCPy concentration. We found that some non-applicators had TCPy levels higher than some applicators. Therefore, the study participants were categorized into high and low exposed groups according to the median TCPy levels across the 3 years of study. In addition to TCPy levels, other exposure variables assessing the duration of exposure were also considered (e.g., years worked in pesticide application, days worked per day, and hours per day). Due to the collinearity among these variables and the others which were not significantly associated with changes in the liver and kidney function, only selected significant exposure variables were considered for the mixed-effects models described below.

Mixed-effects models were used via SAS PROC MIXED procedure to construct longitudinal models to examine the significance of changes in TCPy, cholinesterase activity, and liver and kidney function markers across the application seasons and the cumulative effect of exposure at the end of 3 years of follow-up. TCPy levels were modeled using the 7 timepoints (1, 3, 6, 9, 11, 13, 14), while the cholinesterase activity and the liver and kidney function markers were modeled using 9 timepoints (2, 4, 5, 7, 8, 10, 12, 14, 15). For each measure, different within-subject covariance structures were employed to obtain the best fit model [37]. These structures included Toeplitz, compound symmetry (CS), autoregressive, and unstructured ones. Akaike information criterion (AIC), kikelihood ratio test, and Bayesian information criterion (BIC) were used to obtain the best fit models of each of the previously mentioned structures. CS was the covariate structure that provided the smallest AIC. Scatter and residual plots were used to check for linearity and outliers. 

Exposure and demographic characteristics of participants were considered as candidate predictors of changes in the urinary TCPy, blood cholinesterase activity (AChE, BChE), and serum measures of liver and kidney function (AST, ALT, ALP, GGT, TB, DB, creatinine, and urine) over the application seasons. Time and group as categorical variables were the main variables in the longitudinal regression models. The time variable represents the testing timepoints available for the outcome and the group variable constitutes the high and low exposed groups. Variables with a significant univariate association with the outcome variable were included in the longitudinal models. If the *p*-value of a variable in the model was >0.1, this variable was excluded from the models. Cholinesterase levels as a continuous variable measured at each testing time was considered in the models, but it was not included in any of the models because it was not significantly associated with any of the liver and kidney function measures. Other characteristics of the study sample (e.g., field station and age) were also considered as covariates in the mixed-effects models. All tests of significance were 2-tailed. 

We developed a summary index to examine the clinical impact of exposure to CPF on the liver and kidney function among adolescents. For this summary index, each liver and kidney measure was recoded as either normal (0) or abnormal (1) according to the normal range of each measure [38,39,40]. These variables were then summed to calculate the total number of abnormal tests. This summary index variable was then examined against the group and time variables in a mixed-effects model, including other covariates (e.g., age, field station, and years of working in pesticide application) to assess the changes in the number of clinical abnormal measures over time and between groups.

## 3. Results

### 3.1. Demographic and Exposure Characteristics of the Study Group

Table 1 shows the distribution of the study participants in each field station, their average ages, and the number and percent of pesticide applicators across the 3 years of study. There were comparable numbers of participants from the four field stations (Quesna, Shouhada, Tala, and Berket El-Sabe’) each year, except for the first year, when there were only three field stations. The average age of participants was approximately 16 years in 2014 and increased to about 17 years in 2015 and 2016. We also observed that all participants in 2014 were applicators, while in 2015 and 2016, applicators comprised about 73% of participants in 2015 and 2016. The table presents a detailed description of the different types of pesticide exposure, including working with the MOA, working as private applicators, and application on family farms. In addition, the description of the days of pesticide application in homes for all participants, applicators and non-applicators, was also reported.

Table 2 shows a detailed description of the exposure characteristics of the applicator group, whether they worked with the MOA, mixed/applied as private applicators, mixed/applied in the family farms, or applied at their homes. The data of these characteristics were available at 14 out of the 15 testing timepoints across the 3 years of study, as there was no collection of these data in Time 1. In each year, applicators were tested five times prior to, during, and after the end of the CPF application cycle. The table shows that applicators with the MOA worked for 2 hours on average during the testing days in 2014 and 2016 (Times 2, 12, 13), while in 2015, applicators did not apply with the MOA during the testing days. Across the years of study, applicators with the MOA applied pesticides for 2 days on average during the week prior to testing. The table also presents the number and percent of applicators who worked as private applicators, mixed/applied pesticides in their family farms, or applied pesticides at home. This data shows that, generally, the percentage of applicators who applied pesticide as private applicators, at home, or mixing/applying pesticides on family farms increased during the times of pesticide application by the Ministry of Agriculture (Times 2,3,7,8,12,13).

### 3.2. Exposure and Effect Biomarkers

**Figure 2:**
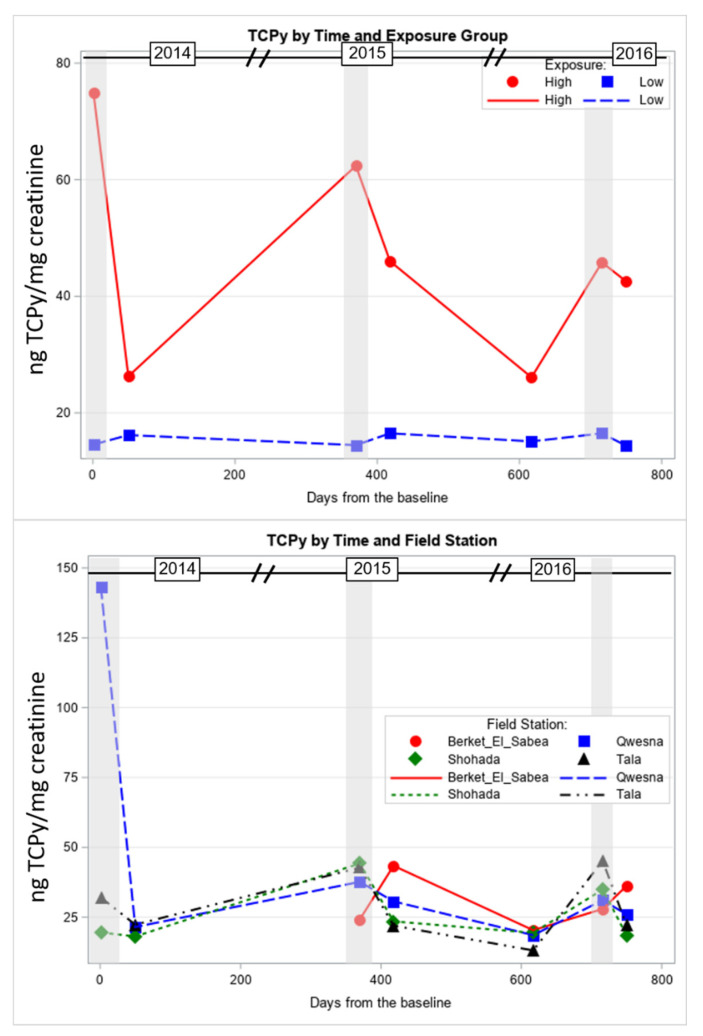
Urinary TCPy levels (least squares mean) in the study participants, stratified by high and low CPF exposure groups and field stations. Shaded areas indicate the times of CPF application.

Modelling urinary TCPy levels across the 3-year study showed spikes of TCPy levels among the high exposed group during CPF application cycles. The average TCPy levels among the high exposed group were triple (in 2014 and 2015) and double (in 2016) the levels in the low exposed group during the CPF application periods (Appendix A). These differences between high and low exposed groups were significant (F = 63.4, *p* < 0.001) across the 3 years of study (Figure 2, Appendix A). In addition, within the high exposure group, there were significant differences in the mean TCPy levels between the timepoints during CPF applications and other times where there was no CPF application (Figure 2, Appendix A). Furthermore, the type of the field station was a significant factor in the TCPy changes across the 3 years of study (F = 2.2, *p* < 0.05). The main difference was that the levels of TCPy among participants from Quesna were the highest at Time 1 (*p* < 0.001). Other differences included significant increases of TCPy among participants from Shohada between Times 2 and 3 and among participants from Tala between Times 5 and 6 (*p* < 0.05) (Figure 2, Appendix A).

**Figure 3:**
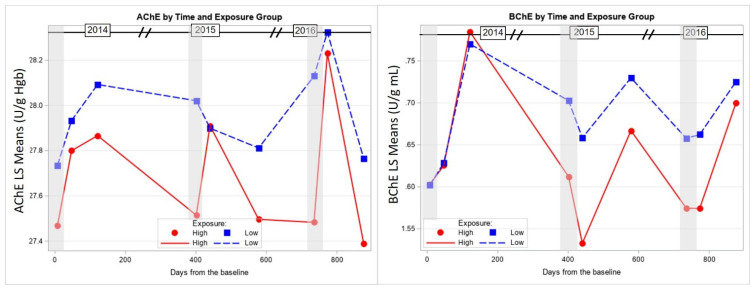
Least squares mean cholinesterase activity (BChE and AChE) of the study group across the 3-year study period stratified by the high and low CPF exposure group. Shaded areas indicate the times of CPF application.

Although time was a significant factor in the changes for both mean blood AChE (F = 5.5, *p* < 0.001) and plasma BChE (F = 13.2, *p* < 0.001) levels across the nine timepoints, BChE activity was more responsive to CPF exposure than that of AChE (Table 3, Figure 3). BChE activity among the high exposed group was inhibited during CPF application and recovered after the end of the application each year. In addition, there were significant differences in mean BChE activity between the high and low exposed groups during the application time in 2015 (Time 5). On the other hand, AChE significantly recovered in 2015 and 2016 after the end of the CPF application without significant differences between high and low exposed groups at any of the nine testing timepoints. We also found that age was a significant predictor for the changes in BChE (f = 5.8, *p* = 0.02), where younger participants showed more BChE inhibition.

### 3.3. Liver and Kidney Functions Changes during the Study

**Figure 4:**
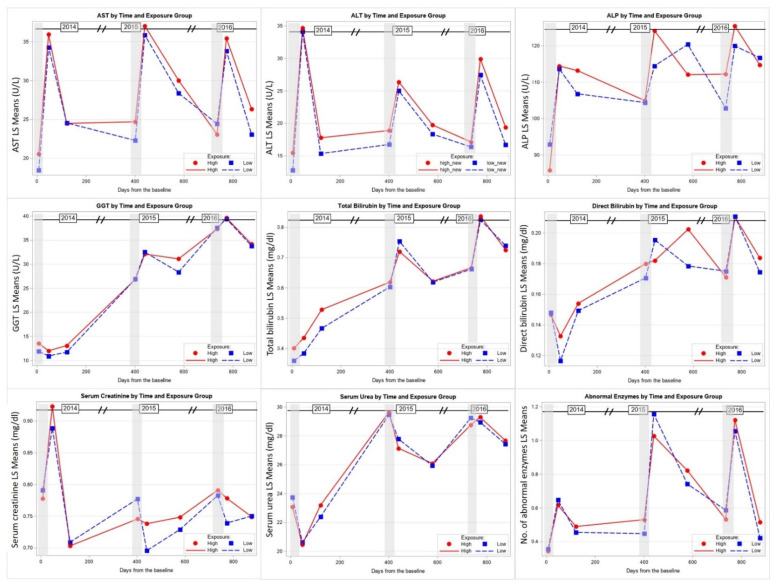
Least squares mean liver and kidney function measures of the study participants across the 3 years of study stratified by the high and low CPF exposure group. Shaded areas indicate the time of CPF application.

Across the 3-year study period, time was a significant factor in the changes for all liver and kidney function measurements (*p* < 0.001). Serum ALT and AST showed a typical pattern across the 3 years of study, with a significant increase following the end of each CPF application cycle (Times 2, 5, 8) and recovery 3 months after the application had ended (Times 3, 6, 9). These changes were observed among both high and low exposed groups. Although there was an overall group difference in serum ALT levels (*p* = 0.02), group differences were not found at any of the nine timepoints. In addition, although there was a significant decrease of the ALT and AST levels 3 months following the end of each application season, the levels did not return to the baseline levels (5 July 2014) at the end of the 3-year study period (Table 4, Figure 4).

Although the other four liver measures (ALP, GGT, TB, DB) showed an increase following the CPF exposure each year (Times 2, 5, 8), the recovery at 3 months following the end of each CPF application cycle (Times 3, 6, 9) was not complete, and the levels continued increasing each year and did not return to the baseline levels (5 July 2014). Toward the end of the 3-year study period, the levels of these measures were 300% (GGT), 200% (TB), and 150% (ALP, DB) of the baseline levels, especially among the high exposed group. The low exposed group showed a similar pattern across the 3 years of study but without significant differences between the two groups. The summary variable of the number of abnormal clinical liver enzyme levels was also tested in a mixed-effects model and showed that there were significant changes over time. The average number of abnormal clinical enzymes increased from an average of 0.6 per person after CPF exposure in 2014 to an average of 1.0 after exposure in 2015 and 2016. This means that, after exposure in 2015 and 2016, each person had an average of one abnormal clinical test following exposure in both years (Table 4, Figure 4).

Serum creatinine levels among both high and low exposed groups showed an increase following the end of the 2014 CPF application cycle (Time 2). Then, serum creatinine levels showed a decrease to the baseline at Time 3 and continued almost at the same level during the following 2 years. Contrary to creatinine, the changes in serum urea levels occurred mainly during 2015 and 2016. The levels showed a significant increase during 2015, with some recovery after the end of application (Times 5 and 6), but the levels significantly increased again during CPF application of 2016. After the end of CPF application in 2016, the levels did not return to the baseline. These changes were similar between both the high and low CPF exposed groups (Table 4, Figure 4).

In addition to the group and time factors, other covariates and moderators, e.g., field station, age, and years of pesticide application, were considered and included in the mixed models for each of the studied measures. Table 3 shows that field station and age were significant factors in the models for most of the studied measures: Age with AST, ALP, total bilirubin, and creatinine and field station with ALT, AST, ALP, and creatinine, while years of pesticide application significantly associated with creatinine changes only. Age and years of pesticide application factors were positively associated with the related measures, where higher levels of liver and kidney function measures were observed in older study participants and those with more years in pesticide application The differences in liver and kidney function measures between the four field stations across the 3 years of study were not conclusive. Table 5 shows that ALT, AST, and ALP serum levels were generally higher among the participants from Quesna and Shohada in comparison to their counterparts from Tala and Berket El-Sabea’. We also found that the highest mean serum creatinine was in Berket El-Sabea’, followed by Shohada and Quesna, while the lowest level was in Tala (Table 5). These findings are generally correlated with the urinary TCPy levels among the filed stations. TCPy levels were higher among the study participants from Quesna at the beginning of the study, then among the participants from Berket El-Sabea’ in the middle of the study. On the other hand, the TCPy levels among the participants from Shohada and Tala were the lowest throughout most of the study (Figure 2, Appendix A Table A1).

## 4. Discussion

The current study characterized longitudinal changes of liver and kidney function among a cohort of pesticide applicators and non-applicators to investigate the effect of repeated exposure to CPF on serum measures of organ function. Liver and kidney function were examined during, immediately following, and approximately 3 months after the end of the CPF application cycle over 3 consecutive years. Although liver and kidney function has been studied among individuals exposed to OP pesticides, the current study has several strengths that have not been previously addressed. The strengths of the current study include the assessment of both a biomarker of CPF exposure (urinary TCPy levels) and biomarkers of effect (liver and kidney function) in relation to the application process to assess the temporal association between both types of biomarkers. In addition, urinary TCPy was assessed quantitatively as a specific biomarker of CPF exposure. Furthermore, the repeated measures design and the mixed models approach employed to analyze the data longitudinally allowed us to use all the available data, examine changes over time while considering other exposure covariates, and investigate the interaction between these covariates.

Our study is unique in identifying the subtle changes in CPF exposure across the 3 years. It confirms our previous findings among the adolescent and adult pesticide applicators in rural Egypt that demonstrated high levels of pesticide exposure [3,30,41,42,43,44]. In a previous study, it was reported that the mean urinary TCPy concentrations among pesticide applicators, engineers, and technicians increased ≥300% once CPF application began compared to their baseline measurements, and the levels remained elevated 8–10 days after the last day of CPF application [30]. Among Egyptian adolescents, TCPy levels in both applicators and non-applicators were elevated during CPF application and reached a maximal level by the end of the CPF application cycle, then recovered 6 months after the end of application season to near baseline levels [42]. When those adolescents were followed up for another season of pesticide application, TCPy levels showed the same pattern as the first season [3]. Cholinesterase activity showed a similar pattern to the changes in urinary TCPy levels, where plasma BChE and blood AChE, mainly BChE, were inhibited during the pesticide application cycle and recovered after the end of application across the 3 years of study. These findings also agree with our previous findings when adolescent pesticide applicators were examined over 10 months in 2010 [42] and when they were followed for another application season in 2011 [3]. In the current work, we were able to replicate these temporal changes of the urinary TCPy levels and blood cholinesterase activity in relation to exposure times. This was available because of the close observation of the application process in the tested field stations and testing participants in relation to the application schedule, which is critical to relate the changes in liver and kidney functions to the exposure status.

Although the average serum measures of the liver and kidney function were in the normal range, the serum measures significantly increased immediately following exposure to chlorpyrifos across the 3 years of study. It is noteworthy to highlight that the changes among both the high and low CPF-exposed groups were parallel, which indicates the ubiquitous exposure among the whole cohort, either occupationally or environmentally. Results also showed that serum AST and ALT were sensitive markers of liver injury in adolescents, increasing directly after CPF application and recovering 3 months after the end of application. On the other hand, serum ALP, GGT, TB, and DB progressively increased each year of the study and did not return to baseline levels. Although the renal functions (serum urea and creatinine levels) were inconsistent across the years of study, urea showed a progressive increase toward the end of the study. Comparing the measured levels to the normal ranges of liver and kidney function, on average, study individuals had one abnormal clinical liver or kidney function serum marker following the 2015 and 2016 application seasons. The average number of abnormal clinical findings of liver and kidney function returned to the baseline at the end of the 3 years of study. The deterioration of liver and kidney function after every application season and their failure to consistently recover at the end of the 3 years of seasonal application are indicators of the cumulative effect of exposure to CPF among those adolescents and suggest long-term deleterious effects of exposure to CPF on the liver and kidney.

Our study is unpreceded in monitoring serum marker of liver and kidney function among pesticide applicators for three consecutive application seasons over 3 years. The majority of the previous studies were cross-sectional in nature and compared the groups only once [11,12,13,14,15,16,21,45,46,47,48,49,50,51,52,53,54], with few studies evaluating the changes in function pre- and post-pesticide exposure [17,20,25,26,54,55]. Even fewer studies have quantitatively assessed the pesticide exposure [50,53,55]. Designing a prospective longitudinal study allowed us to track the liver and kidney functions before and after three consecutive application seasons and record the changes as a result of the exposure. 

Chronic kidney disease among young males working in agriculture was reported. We observed a statistically significant increase of the serum urea levels among our study cohorts in 2015 and 2016 without a return to baseline levels, which may be an early indicator for CKD. Our results are consistent with those found among agricultural workers in Central American studies [20,25,26]. In these studies, the repeated exposure of farmworkers led to progressive renal dysfunction during the planting season [20,25] and toward the end of the harvest season [25]. One-third of the farmworkers who experienced a recent increase in their renal functions were diagnosed with CKD [20]. In addition, Hu and colleagues examined the effect of acute pesticide exposure on kidney function within 3 days of exposure. However, the study design was not able to assess the cumulative effect of exposure, as it investigated the function one time in the 4–10 days following exposure and there was no study of the repeated exposure [17].

Oxidative stress is one mechanism that may contribute to the toxicity of pesticides, leading to changes in metabolic function and ultimately resulting in cell death [55]. A noncholinergic, pro-oxidant mechanism for CPF and chlorpyrifos-oxon (CPF-O)-induced toxicity was recently investigated by Naime et al. [56]. In vitro CPF and CPF-O exposures resulted in significantly reduced levels of antioxidant glutathione that preceded a significant decrease in neuronal cell viability. This study shows that, in addition to being a more potent AChE inhibitor, CPF-O is also a more potent pro-oxidant molecule when compared with CPF, highlighting the role of CPF metabolism (bioactivation to CPF-O) in the ensuing noncholinergic toxicity. Thus, oxidative injury could contribute to liver and kidney damage since the human liver and kidney both express CYP2B6, the enzyme primarily responsible for the bioactivation of CPF to CPF-O [5,6]. In addition, subacute, low-dose exposure to OP pesticides that form toxic oxon metabolites has been reported to produce hyalinization, vacuolization, nucleus necrosis, hepatocellular edema, and fatty degeneration [8,57]. These morphological changes could also be associated with a disruption of cell function which may be related to lower antioxidative capability, changes in fatty and glycogen content in the liver, and inhibition of some enzymes contributing to lipid, carbohydrate, and protein metabolism important to preserve the integrity of the liver tissue [55]. Coupling these mechanisms with individual susceptibility as a result of genetic, environmental, or biological factors may lead to an increased risk of developing chronic effects on liver and kidney [16].

Different determinants of exposure were also studied to examine their relationship with the liver and kidney function among the study participants. The final models included, in addition to high and low CPF exposure group and time, one or more of the following significant predictors: Field station, age, and years of working in pesticide application. Whereas the relationship of age and years of application with the liver and kidney measures was straightforward, the differences between field stations in the liver and kidney functions were not conclusive. The current study showed that serum markers of liver and kidney function deteriorated more among participants of older ages or those with more years working in pesticide application, which is similar to the greater renal dysfunction observed among older agricultural farmworkers in El Salvador [21]. In our previous work with a similar group of pesticide applicators, we also reported that biochemical parameters, e.g., hemoglobin levels, serum ALT, ALP, and basophils number, were positively correlated with days worked the current season or years worked in pesticide application [54]. In another cohort of pesticide applicators in the same area in Egypt, total hours applying CPF and total hours applying other pesticides in the field were the strongest predictors of cumulative urinary TCPy, while the duration of time applying pesticides prior to blood draw was the strongest predictor of peak TCPy [41]. In the same study, although wearing personal protective equipment (PPE) was associated with lower urinary TCPy levels, it was unlikely that the participants reported using the appropriate PPE [41]. Other studies have presented evidence of liver damage among workers in pesticide manufacturing after their exposure to high levels of OP or to low levels of these pesticides for a longer period of time or among workers who did not comply with the proper use of PPE [27].

Despite the many strengths of our study, it still faces some challenges. First, urinary TCPy measurements were not available at all timepoints of the liver and kidney function measurements, which did not allow us to include the TCPy levels as a continuous variable in the mixed-effects model. Second, the application schedule and biological assessments at the four field stations of the study were not identical. Although the application schedule was generally similar across the field stations, there have been a few days difference between them. For logistical reasons, testing occurred over a 4-day period, as we tested one field station per day. For the sake of modelling the exposure and the outcome variables, we assumed that the pesticide application and testing schedules were the same in all field stations. Third, a control group was not available where our non-applicator group showed increased levels of both urinary TCPy and serum markers of liver and kidney function, parallel to the levels among applicators. Nevertheless, finding such a reference group without exposure to pesticides in rural Egypt is not an easy task given the widespread exposure to pesticides from different sources, e.g., food, water, home application, and drift from fields to the surrounding areas. Another limitation of the study is the absence of a non-applicator group in 2014. We overcame this challenge by categorizing the total study group into high and low CPF exposure groups in using the median urinary TCPy levels across the 3 years of study as a cut-off point. 

## 5. Conclusions

The study showed that the frequent assessment of serum measures of liver and kidney function is a sound tool to evaluate the repeated injury of the liver and kidney associated with chronic repeated occupational and environmental exposure to chlorpyrifos. Results showed that, although some liver function parameters, e.g., serum ALT and AST, were able to recover at the end of each application season, other liver measures, e.g., serum ALP, GGT, TB, and DB, as well as serum urea levels, showed progressive increases each year. The final levels of these measures failed to return to their respective baselines at any time during the 3-year study period. Comparing serum measures of liver and kidney function to the range of normal values, an average of one abnormal serum marker was observed in the participants during the 2015 and 2016 seasons. Together, the outcomes of this study support the need to continuously follow up workers exposed to OP pesticides to detect elevated serum markers of liver and kidney function before the development of clinical health effects.

## Figures and Tables

**Table 1:** Distribution and demographic characteristics of the study participants across the 3 years of study (from baseline questionnaires).

Demographic Characteristics		Study Year		
		**2014** **(*n* = 119)**	**2015** **(*n* = 279)**	**2016** **(*n* = 249)**
Field stations	Quesna, No. (%)	44 (37.0)	79 (28.3)	69 (27.7)
	Shohada, No. (%)	44 (37.0)	74 (26.5)	71 (28.5)
	Tala, No. (%)	31 (26.0)	63 (22.6)	57 (22.9)
	Berket El-Sabe’, No. (%)	-	63 (22.6)	52 (20.9)
Age	Mean ± SD	16.1 ± 1.8	16.5 ± 2.6	17.4 ± 2.7
Applicators	No. (%)	119 (100.0)	203 (72.8)	182 (73.1)
Applying with the Ministry of Agriculture	No. *	87	139	130
	Years worked:			
	Range	1–6	1–7	2–8
	Mean ± SD	2.4 ± 1.4	3.0 ± 1.3	4.1 ± 1.4
	Days/week:			
	Range	1–6	1–6	1–5
	Mean ± SD	3.0 ± 1.1	2.7 ± 1.0	2.7 ± 0.7
	Hours/day:			
	Range	1–6	1–7	1–5
	Mean ± SD	3.7 ± 1.1	2.8 ± 1.2	2.8 ± 0.8
Applying as private applicator	No. *	45	41	43
	Times/week:			
	Range	1–6	1–4	1–4
	Mean ± SD	2.8 ± 1.4	2.3 ± 0.7	2.4 ± 0.7
Applying in family farm	No. *	88	133	116
Application at home (numbers among all participants)	No.	96	96	249
	Times in the past year:			
	Range	2–365	1–70	5–85
	Mean ± SD	31.8 ± 40.0	23.1 ± 12.1	31.4 ± 12.8

* The numbers are not exclusive.

**Table 2:** Exposure characteristics of the pesticide applicator group at each testing timepoint across the 3 years of study (from the repeated questionnaire). Shaded areas indicate the times of CPF application. There was no collection of these data at Time 1.

Study Year		2014				2015					2016				
**Testing Time (Date)**		Time 2 (Jul 5)	Time 3 (Jul 12)	Time 4 (Jul 19)	Time 5 (Oct 25)	Time 6 (Jul 12)	Time 7 (Jul 25)	Time 8 (Aug 15)	Time 9 (Aug 29)	Time 10 (Dec 12)	Time 11 (Apr 16)	Time 12 (Jul 9)	Time 13 (Jul 23)	Time 14 (Aug 27)	Time 15 (Dec 10)
**Day from Baseline**		7	14	21	121	388	401	4221	435	58	672	735	749	773	876
**Total No. (%) of applicators**		118 (100.0)	106 (100.0)	104 (100.0)	85 (100.0)	129 (74.6)	153 (72.9)	150 (71.1)	153 (72.9)	152 (75.6)	148 (74.0)	158 (72.8)	156 (75.0)	141 (73.4)	146 (73.0)
**MOA**	Hours today: No. Range Mean ± SD	51 1.0–4.0 2.1 ± 0.5	0	0	0	0	0	0	0	0	0	7 2.0–2.0 2.0 ± 0.0	41 2.0–2.5 2.2 ± 0.2	0	0
	Days/week in the past week: No. Range Mean ± SD	31 1–5 1.6 ± 1.0	8 1–3 1.9 ± 0.6	8 1–3 1.9 ± 0.6	0	90 1–2 1.9 ± 0.3	108 1–2 1.9 ± 0.3	102 1–3 1.8 ± 0.6	0	0	0	113 1–3 1.9 ± 0.3	32 1 1.0 ± 1.0	0	0
**Mixed/applied as private in the past week**	No. (%)	1 (0.8)	8 (7.5)	3 (2.9)	0	28 (21.7)	21 (13.7)	13 (8.7)	15 (9.8)	6 (3.9)	0	18 (11.4)	6 (3.8)	15 (10.6)	6 (4.1)
**Applied at home in the past week**	No. (%)	12 (10.2)	17 (16.1)	3 (2.9)	0	102 (79.1)	138 (90.2)	2 (1.3)	4 (2.6)	0	83 (56.1)	109 (69.0)	54 (34.6)	4 (2.8)	44 (30.1)
**Mixed/applied in the family farm in the past week**	No. (%)	6 (5.1)	6 (5.7)	7 (6.7)	0	80 (62.0)	93 (60.8)	47 (31.3)	60 (39.2)	26 (17.1)	4 (2.7)	37 (23.4)	15 (9.6)	40 (28.4)	28 (19.2)

**Table 3:** Cholinesterase activity (least squares mean ± SE) across the 3 years of study and the relationship with the covariates of time, group, and the interaction of time and group. For BChE, age was a significant covariate (f = 5.8, *p* = 0.02). Cholinesterase activity was assessed at 9 timepoints during the 3-year period. Shaded areas indicate the times of CPF application.

Study Year		2014			2015			2016			Time	Group	T*G
**Testing Time**		**2**	**4**	**5**	**7**	**8**	**10**	**12**	**14**	**15**			
**AChE** **(U/g Hgb)**	High exposed	27.5 ± 0.3	27.8 ± 0.3	27.9 ± 0.4	27.5 ± 0.3	**27.9 ± 0.3 ^b^**	**27.5 ± 0.3 ^b^**	27.5 ± 0.3	**28.2 ± 0.3 ^ab^**	**27.4 ± 0.3 ^b^**	5.5, <0.001	0.5, 0.9	1.4, 0.2
	Low exposed	27.7 ± 0.3	27.9 ± 0.3	28.1 ± 0.4	28.0 ± 0.3	27.9 ± 0.3	27.8 ± 0.3	28.1 ± 0.3	**28.3 ± 0.3 ^a^**	**27.8 ± 0.3 ^b^**			
	Group Diff.												
**BChE** **(U/mL)**	High exposed	1.6 ± 0.05	1.6 ± 0.03	**1.8 ± 0.05 ^ab^**	**1.6 ± 0.04 ^b^**	**1.5 ± 0.04 ^b^**	**1.7 ± 0.04 ^b^**	1.6 ± 0.04	1.6 ± 0.04	**1.7 ± 0.04 ^ab^**	13.2, <0.001	1.0, 0.3	2.0, 0.04
	Low exposed	1.6 ± 0.05	1.6 ± 0.05	**1.08 ± 0.05 ^ab^**	**1.7 ± 0.04 ^a^**	1.7 ± 0.05	**1.7 ± 0.04 ^a^**	1.7 ± 0.04	1.7 ± 0.04	**1.7 ± 0.04 ^ab^**			
	Group Diff.					**^c^**							

- ^a^ Significantly different from baseline; ^b^ significantly different from the previous timepoint; ^c^ significant difference between high and low CPF exposure groups.

**Table 4:** Liver and kidney function measures (least squares mean ± SE) of the high and low CPF exposure groups using repeated mixed-effects models and the relationship with the covariates of time, group, and the interaction of time and group.

Study Year		2014			2015			2016			Time	Group	T*G
**Testing Time**		**2**	**4**	**5**	**7**	**8**	**10**	**12**	**14**	**15**			
**ALT (U/L)**	High exposed	15.5 ± 1.4	**34.7 ± 1.5 ^ab^**	**17.8 ± 1.7 ^b^**	**18.9 ± 0.9 ^a^**	**26.4 ± 1.0 ^ab^**	**19.8 ± 1.0 ^ab^**	**17.2 ± 0.9 ^b^**	**29.9 ± 1.0 ^ab^**	**19.4 ± 1.0 ^ab^**	69.2, <0.001	6.0, 0.02	0.3, 1.0
	Low exposed	12.8 ± 1.4	**34.1 ± 1.5 ^ab^**	**15.4 ± 1.5 ^b^**	**16.8 ± 1.0 ^a^**	**25.0 ± 1.0 ^ab^**	**18.4 ± 1.0 ^ab^**	**16.4 ± 0.9 ^a^**	**27.5 ± 1.0 ^ab^**	**16.7 ± 1.0 ^ab^**			
	Group Diff.												
**AST (U/L)**	High exposed	20.9 ± 1.8	**36.2 ± 1.9 ^ab^**	**24.8 ± 2.1 ^b^**	**24.9 ± 1.2 ^a^**	**37.3 ± 1.2 ^ab^**	**30.0 ± 1.2 ^ab^**	**22.9 ± 1.1 ^b^**	**35.3 ± 1.2 ^ab^**	**26.2 ± 1.2 ^ab^**	44.7, <0.001	3.3, 0.07	0.7, 0.7
	Low exposed	18.7 ± 1.7	**34.5 ± 1.8 ^ab^**	**24.8 ± 1.8 ^ab^**	**22.3 ± 1.2**	**35.8 ± 1.2 ^ab^**	**28.4 ± 1.2 ^ab^**	**24.1 ± 1.2 ^ab^**	**33.5 ± 1.6 ^ab^**	**22.7 ± 1.2 ^ab^**			
	Group Diff.									*			
**ALP (U/L)**	High exposed	85.8 ± 8.3	**114.4 ± 8.9 ^ab^**	**113.2 ± 9.7 ^a^**	**104.9 ± 5.3 ^a^**	**124.2 ± 5.6 ^ab^**	**112.1 ± 5.5 ^a^**	**112.2 ± 5.3 ^a^**	**125.5 ± 5.5 ^a^**	**114.8 ± 5.5 ^a^**	4.4, <0.001	0.2, 0.65	0.6, 0.74
	Low exposed	93.0 ± 7.8	113.6 ± 8.5	106.8 ± 8.7	104.2 ± 5.4	**114.5 ± 5.6 ^a^**	**120.4 ± 5.8 ^a^**	**102.9 ± 5.4 ^b^**	**120.0 ± 5.7 ^ab^**	**116.7 ± 5.7 ^a^**			
	Group Diff.												
**GGT (U/L)**	High exposed	13.6 ± 1.6	12.0 ± 1.7	13.1 ± 1.9	**26.9 ± 1.0 ^ab^**	**32.1 ± 1.1 ^ab^**	**31.1 ± 1.0 ^a^**	**37.4 ± 1.0 ^ab^**	**39.7 ± 1.1 ^a^**	**34.2 ± 1.1 ^ab^**	128.1, <0.001	1.4, 0.2	0.4, 0.9
	Low exposed	11.9 ± 1.5	11.0 ± 1.6	11.7 ± 1.7	**26.9 ± 1.0 ^ab^**	**32.5 ± 1.1 ^ab^**	**28.4 ± 1.1 ^ab^**	**37.6 ± 1.0 ^ab^**	**39.5 ± 1.1 ^a^**	**33.8 ± 1.1 ^ab^**			
	Group Diff.												
**Total Bilirubin (mg/dl)**	High exposed	0.40 ± 0.03	0.44 ± 0.03	**0.53 ± 0.04 ^ab^**	**0.62 ± 0.02 ^ab^**	**0.72 ± 0.02 ^ab^**	**0.62 ± 0.02 ^ab^**	**0.67 ± 0.02 ^a^**	**0.84 ± 0.02 ^ab^**	**0.73 ± 0.02 ^ab^**	68.4, <0.001	1.3, 0.3	0.7, 0.8
	Low exposed	0.36 ± 0.03	0.38 ± 0.03	**0.47 ± 0.03 ^a^**	**0.60 ± 0.02 ^ab^**	**0.75 ± 0.02 ^ab^**	**0.62 ± 0.02 ^b^**	**0.66 ± 0.02 ^a^**	**0.82 ± 0.02 ^ab^**	**0.74 ± 0.02 ^ab^**			
	Group Diff.												
**Direct Bilirubin (mg/dl)**	High exposed	0.15 ± 0.01	0.13 ± 0.01	0.15 ± 0.01	**0.18 ± 0.01 ^a^**	**0.18 ± 0.01 ^a^**	**0.20 ± 0.01 ^a^**	**0.17 ± 0.01 ^b^**	**0.21 ± 0.01 ^ab^**	**0.18 ± 0.01 ^ab^**	12.7, <0.001	1.0, 0.3	0.9, 0.5
	Low exposed	0.15 ± 0.01	0.12 ± 0.01	0.15 ± 0.01	**0.17 ± 0.01 ^b^**	**0.19 ± 0.01 ^ab^**	**0.18 ± 0.01 ^a^**	**0.18 ± 0.01 ^a^**	**0.21 ± 0.01 ^ab^**	**0.17 ± 0.01 ^b^**			
	Group Diff.						c						
**Creatinine (mg/dl)**	High exposed	0.78 ± 0.02	**0.92 ± 0.03 ^ab^**	**0.70 ± 0.03 ^ab^**	0.75 ± 0.02	0.74 ± 0.02	0.75 ± 0.02	0.79 ± 0.02	0.78 ± 0.01	0.75 ± 0.02	12.4, <0.001	0.6, 0.4	0.9, 0.5
	Low exposed	0.79 ± 0.02	**0.89 ± 0.03 ^ab^**	**0.71 ± 0.03 ^ab^**	**0.78 ± 0.02 ^b^**	**0.70 ± 0.02 ^ab^**	**0.73 ± 0.02 ^a^**	0.78 ± 0.02	0.74 ± 0.02	0.75 ± 0.02			
	Group Diff.												
**Urea (mg/dl)**	High exposed	23.1 ± 1.0	20.5 ± 1.1	23.3 ± 1.1	**29.6 ± 0.7 ^ab^**	**27.1 ± 0.7 ^ab^**	**26.0 ± 0.7 ^a^**	**28.9 ± 0.6 ^ab^**	29.4 ± 0.7	**27.8 ± 0.7 ^a^**	28.1, <0.001	0.3, 0.6	0.2, 1.0
	Low exposed	24.0 ± 0.9	**20.9 ± 1.0 ^ab^**	22.6 ± 1.0	**29.7 ± 0.7 ^ab^**	**28.1 ± 0.7 ^a^**	**26.2 ± 0.7 ^ab^**	**29.7 ± 0.6 ^ab^**	**29.4 ± 0.7 ^a^**	**27.8 ± 0.7 ^a^**			
	Group Diff.												
**Summary Index**	High exposed	0.3 ± 0.1	0.6 ± 0.1	0.5 ± 0.1	0.5 ± 0.1	**1.0 ± 0.1 ^ab^**	**0.8 ± 0.1 ^a^**	**0.5 ± 0.1 ^b^**	**1.1 ± 0.1 ^ab^**	**0.5 ± 0.1 ^b^**	16.3, <0.001	0.7, 0.4	0.4, 0.9
	Low exposed	0.3 ± 0.1	0.6 ± 0.1	0.4 ± 0.1	0.4 ± 0.1	**1.1 ± 0.1 ^ab^**	**0.7 ± 0.1 ^ab^**	0.5 ± 0.1	**1.0 ± 0.1 ^ab^**	**0.4 ± 0.1 ^b^**			
	Group Diff.												

- Shaded areas indicate the times of CPF application. - These are 9 out of the 15 timepoints of the study where liver and kidney function was assessed - All estimates are from linear mixed-effects models that included in addition to time, group, time*group (T*G): For ALT, field station was a significant covariate (f = 2.7, *p* = 0.0495); For AST, field station (f = 10.6, *p* < 0.001) and age (f = 5.0, *p* = 0.03) were significant covariates; For ALP, field station (f = 3.4, *p* = 0.02) and age (f = 35.1, *p* < 0.001) were significant covariates; For total bilirubin, age was a significant covariate (f = 4.5, *p* = 0.03); For creatinine, field station (f = 2.2, *p* = 0.09), age (f = 14.6, *p* < 0.001), and years of pesticide application (f = 4.9, *p* = 0.03) were significant covariates; For the summary index, field station was a significant covariate (f = 3.8, *p* = 0.01). - ^a^ Significant difference with baseline; ^b^ significant difference with the previous timepoint; ^c^ significance between high and low CPF exposure groups, based on urinary TCPy levels.

**Table 5:** Main effects of liver and kidney function measures (least squares mean ± SE) of the study participants at the 4 field stations and the significant difference from the repeated mixed-effects models.

Liver & Kidney Function	Field Stations				Notes
	Berket El-Sabea’	Quesna	Shohada	Tala	
AST (U/L)	24.5 ± 1.0	30.7 ± 0.8	29.7 ± 0.7	27.0 ± 0.9	Quesna and Shohada were significantly higher than Berket El-Sabea’ and Tala
ALT (U/L)	19.4 ± 0.9	21.7 ± 0.7	22.4 ± 0.6	21.5 ± 0.8	Quesna and Shohada were significantly higher than Berket El-Sabea’
ALP (U/L)	100.7 ± 4.2	114.3 ± 3.1	115.8 ± 2.9	113.6 ± 3.7	Quesna, Shohada, and Tala were significantly higher than Berket El-Sabea’
Creatinine (U/L)	0.79 ± 0.02	0.77 ± 0.01	0.77 ± 0.01	0.74 ± 0.01	There were significant differences between the 4 field stations

## Data Availability

The data presented in this study are available on request from the corresponding author. The data are not publicly available due to privacy and ethical concerns.

## Appendix A

**Table A1:** Urinary TCPy levels (ng TCPy/mg creatinine, least squares mean ± SE) at the 7 timepoints where TCPy was assessed, stratified by high and low CPF exposure groups and field stations. Shaded areas indicate the time of CPF application.

Study Year		2014		2015		2016			Time	Group	T*G
**Testing Time**		**1**	**3**	**6**	**9**	**11**	**13**	**14**			
**Field Station**	**Quesna**	143.3 ± 12.2	**21.5 ± 10.1 ^a^**	**37.7 ± 9.4 ^a^**	**30.7 ± 8.5 ^a^**	**18.6 ± 8.4 ^a^**	**31.1 ± 8.2 ^a^**	**26.0 ± 8.5 ^a^**	8.5, <0.001	2.2, <0.05	5.2, <0.001
	**Shohada**	19.6 ± 10.3	18.1 ± 9.8	**44.3 ± 8.4 ^ab^**	**23.5 ± 7.9 ^b^**	19.4 ± 8.5	34.9 ± 8.5	18.6 ± 8.6			
	**Tala**	32.1 ± 11.7	22.4 ± 12.6	42.8 ± 9.1	22.0 ± 9.5	13.1 ± 10.3	**45.2 ± 9.9 ^b^**	22.1 ± 10.2			
	**Berket El-Sabea’**			24.3 ± 14.3	43.4 ± 10.5	20.4 ± 9.6	28.0 ± 9.8	36.2 ± 0.2			
	**Group Diff.**	c									
**TCPy**	**High exposed**	74.9 ± 7.3	**26.3 ± 8.0 ^a^**	**62.4 ± 6.3 ^b^**	**46.0 ± 6.3 ^ab^**	**26.1 ± 7.5 ^ab^**	**45.8 ± 5.3 ^b^**	42.6 ± 6.8	3.0, 0.007	63.4, <0.001	3.0, 0.007
	**Low exposed**	14.6 ± 11.0	16.2 ± 8.0	**14.5 ± 6.6 ^a^**	16.5 ± 5.5	15.1 ± 5.2	16.5 ± 6.6	**14.4 ± 5.6 ^a^**			
	**Group Diff.**	c		c	c		c	c			

^a^ Significant difference with baseline; ^b^ significant difference with the previous timepoint; ^c^ significance between high and low CPF exposure groups, based on urinary TCPy levels.
